# Medicines Administered by the Nose

**Published:** 1844-05

**Authors:** 


### SELECTIONS

FROM

### AMERICAN AND FOREIGN JOURNALS.

---

*Medicines Administered by the Nose.*—Dr. Wolford Nelson reports the following cases in the Boston Medical and Surgical Journal of the 27th March, in which medicines were administered by the nose after the loss of the power of deglution:

"A stout, plethoric girl was taken with convulsions, shortly after a full meal at supper. The family physician was called in, but the convulsions were so violent, and the jaws so firmly locked, that he could not introduce any medicine into the mouth. Attempts at bleeding were made; frictions, sinapisms, sprinkling of cold water, &c., were used, to no purpose. At 11, P. M., I was called in. Never had I seen a more violent case of hysterical convulsions—the friends apprehended speedy death. Twenty grains of pulv. ipecac. with 1 gr. potassio-tart. ant. were mixed in a spoonful of water. This was poured into the nostrils, and passed down, as was manifest by the action of the throat. Immediately after, ʒss. spt. ammon. arom., undiluted, was sent after the other; this produced a marked uneasiness; sneezing followed, but soon ceased. The fits returned, and the aromatic spirit was repeated, at least ten or a dozen times. The face gradually lost its livid hue, the patient became more tranquil, and in twenty minutes the 'jaw fell,' and copious vomiting of much acid and undigested food followed. The next morning my patient was up and about, to the astonishment of all, and well, save a tenderness in the nose and some heat in the fauces.

"Not long after the occurrence of the preceding case, I was requested to see a fine, and most completely 'spoiled child,' four years old. Speedy vomiting seemed to be indicated; 10 grs. pulv. ip. and gr. i. of tart. ant. were repeatedly forced into the mouth, and as often spurted out in the faces of the assistants—holding the head back, and ample pinching of the nose, notwithstanding. Seeing this, a similar dose was prepared in a spoonful of water, and poured into the nostril. This readily passed into the stomach, maugre the efforts the urchin made to send it whence it came. The medicine had the desired effect, and, I take it, saved the wayward child. After this, the little vixen was ready enough to take medicines in the right way.

"Last summer I was called in haste to a sailor, who had fallen into the river, and had been under water 'upwards of ten minutes,' as affirmed by the captain and by-standers. He had all the appearance of being dead—the face bloated and livid, the mouth filled with froth and mucus. Another practitioner had preceded me, and was industriously occupied in rubbing the body, which was cold and exposed to the air. He was immediately wrapped up in warm blankets, and, under these, dry mustard was abundantly rubbed over the whole surface, while I was busily employed with the spt. ammon. arom., first pouring ʒss. down the nostril, then dipping a quill, saturated with the *aqua ammonia*, and which was thrust down the nose as far as could be reached. This caused some motion—the face became a little florid, a feeble attempt at sneezing was evident—then an attempt to cough, but in a moment after, all was again still; but by persevering in this course for fifteen minutes, the man sneezed forcibly. From this instant it was easy to produce excitement. Thirty grains of pulv. ipecac., mixed with water, were now poured down the nose. In about ten minutes, vomiting occurred; much mucus, and the remains of a half-digested dinner, came up. In one hour he was partly conscious; he was then bled ℥xvi., followed by suitable aperients, &c., and in a few days, to use his own language, he 'would be quite well, if it was not for the infernal burning and itching of the skin [caused by the mustard] and the thump on the head,' for he struck it on a plank while falling into the water.

"A few months since, I was called to a man stated to be dying, from the effects of an extraordinary portion of whiskey he had just taken. He was cold and clammy; the face and the extremities quite blue; mouth filled with froth; breathing nearly suspended, and pulse countless. He had all the appearance of one in *articulo mortis* with Asiatic cholera. Not having a stomach-pump at hand, and not having time to wait for one, as life was nearly gone, 30 grs. of pulv. ipecac. were at once mixed up and poured down the nose, as nothing could be passed through the mouth; and the slight effort to swallow led me to think it was trickling down the throat. Immediately after, ℥ss. spt. ammon. aro. was poured into the other nostril; this caused manifest uneasiness, but nothing more. Another portion was administered, which produced some wincing. A long quill, well saturated with the common aqua ammonia, was repeatedly thrust in through the nose. An effort to sneeze, then a cough, and then a good hearty sneeze, assured me that the toper was not entirely gone. Nausea soon became apparent, vomiting followed, and in two hours, the wretched man had knowledge enough to ask where he was, and the next day was sufficiently well to take the temperance pledge.

"An athletic man, raving mad with *delirium tremens*, to whom it was impossible to give any medicine by the mouth, was in two hours in a state of tranquility, having *swallowed* through the nose one grain ext. belladonna, two grains of pulv. ipecac., and three grains pulv. opii, mixed with water in a spoon."

